# Supplementary material for: Causal Relationship between Aging and Anorexia Nervosa: A White-Matter-Microstructure-Mediated Mendelian Randomization Analysis
Source: Biomedicines. 2024 Aug 16;12(8):1874. doi: 10.3390/biomedicines12081874 (PMC11351342; doi:10.3390/biomedicines12081874)
Supplement: Supplementary file 1 [file biomedicines-12-01874-s001.zip › Supplementary Figure/Figure S8/Leaveoneout plot of MR analysis for TL to ubm-b-1586.pdf]

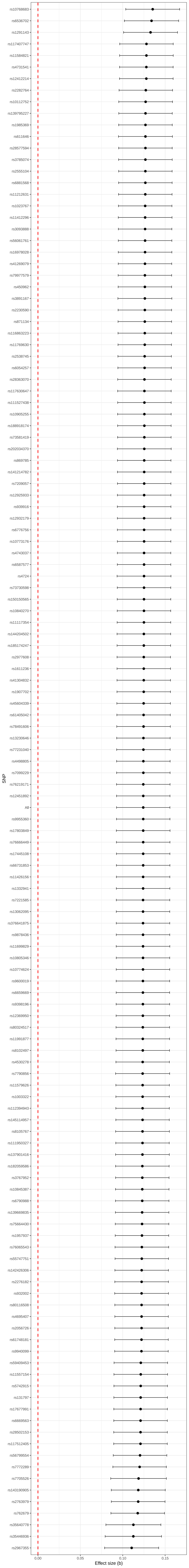

rs10768683
rs6536702
rs1291143
rs117407747
rs11584821
rs4731541
rs12412214
rs2282764
rs10112752
rs139795227
rs1985369
rs611646
rs28577594
rs3785074
rs2555104
rs6881568
rs11212631
rs1023767
rs11412296
rs3093888
rs56061761
rs16978028
rs41269079
rs79977579
rs450962
rs3891167
rs2230590
rs871134
rs116863223
rs11769630
rs2538745
rs6054257
rs28363070
rs117630647
rs111527438
rs10905255
rs188918174
rs73581419
rs202034370
rs869785
rs141214782
rs7209057
rs12925933
rs939916
rs12932179
rs6776756
rs10773176
rs4743037
rs6587577
rs4724
rs73730598
rs150150565
rs10840270
rs11117354
rs144204502
rs185174247
rs2977608
rs1611236
rs41304832
rs1907702
rs45604339
rs61405042
rs78491606
rs13230646
rs77231040
rs4498805
rs7099229
rs76219171
rs12451892
A8
rs9955360
rs17803849
rs76666449
rs17445108
rs66731853
rs11426156
rs1332941
rs7221585
rs13062095
rs376641875
rs9878436
rs11699829
rs10805346
rs10774624
rs9600019
rs6659669
rs9398196
rs12369950
rs80324517
rs11991877
rs8102497
rs4530278
rs7790856
rs11579626
rs1003322
rs112394943
rs145114957
rs8105767
rs111950327
rs137901418
rs182059566
rs3767952
rs10845387
rs6790988
rs139669835
rs75664430
rs1957937
rs76065543
rs55747751
rs142426306
rs2276182
rs932002
rs80116508
rs4695407
rs2056726
rs61748181
rs9940099
rs59409453
rs11557154
rs5742915
rs131797
rs17677991
rs6669563
rs28502153
rs117512405
rs56799554
rs7772289
rs7705526
rs143190905
rs2763979
rs762679
rs35640778
rs35446936
rs2967355
SNP
0.00
0.05
0.10
0.15
Effect size (b)
